# Major Depressive Disorder and Stroke Risks: A 9-Year Follow-Up Population-Based, Matched Cohort Study

**DOI:** 10.1371/journal.pone.0046818

**Published:** 2012-10-08

**Authors:** Cheng-Ta Li, Ya-Mei Bai, Pei-Chi Tu, Ying-Chiao Lee, Yu-Lin Huang, Tzeng-Ji Chen, Wen-Han Chang, Tung-Ping Su

**Affiliations:** 1 Department of Psychiatry, Taipei Veterans General Hospital, Taipei, Taiwan; 2 Institute of Brain Science, National Yang-Ming University, Taipei, Taiwan; 3 Division of Psychiatry, Faculty of Medicine, National Yang-Ming University, Taipei, Taiwan; 4 Department of Dermatology, Faculty of Medicine, National Yang-Ming University, Taipei, Taiwan; 5 Department of Family Medicine, Taipei Veterans General Hospital, Taipei, Taiwan; University of Sydney, Australia

## Abstract

**Background and Purpose:**

Major depressive disorder (MDD) is characterized by recurrent depressive episodes and one of the treatment choices is antidepressants. Patients with MDD are at greater risk of developing major metabolic diseases that may in turn lead to stroke. Moreover, both depressive symptoms and taking antidepressant medications are associated with higher risk of stroke. However, whether and how clinical depression increases stroke risk remains an unanswered question. Our aim was to provide answers to this question.

**Methods:**

A matched cohort study of 5015 subjects (1003 MDD patients and 4012 control subjects) was conducted using a nationwide database. Subjects were followed to a maximum of 9 years to determine rates of newly-developed strokes, and controls and MDD groups with different levels of antidepressant refractoriness were compared to determine the temporal relation between stroke and three major metabolic comorbidities (i.e., diabetes mellitus, hypertension and hyperlipidemia). The levels of depressive symptoms and the antidepressant medications before stroke onset were investigated.

**Results:**

Patients with MDD had significantly higher rates of stroke (4.3% vs. 2.8%, p<0.05) during the follow-up. Mediation regression analyses revealed that the occurrence of stroke in the MDD subjects was significantly mediated by the development of major metabolic diseases. Greater severity of depression, but not greater use of antidepressants, preceded the occurrence of stroke.

**Conclusions:**

A clinical diagnosis of major depression leads to stroke indirectly through more intense depressive symptoms and the development of major comorbidities.

## Introduction

Stroke is a leading cause of morbidity and mortality worldwide [1]. The most frightening aspects of stroke are its sudden onset and the residual mental or physical after-effects that negatively impact the rest of life [2], [3].

The reported risk factors for stroke include hypertension (HTN), obesity/hyperlipidemia, diabetes mellitus, substance abuse (e.g., alcoholism), and old age [3]–[8]. Studies suggest that depression is another significant risk factor for stroke [9], [10]. However, some limitations exist in the interpretation of the depression-stroke association in the general population. Firstly, most studies assess depression based on self-reported questionnaires using depression rating scales rather than on clinical diagnoses of depression [9], [10]. Depressive symptoms that occasionally (e.g., under stressful situations) occur in healthy subjects are different from a clinical diagnosis of depression, which leads to prominent functional impairments and requires treatment (e.g., by antidepressant drugs). Secondly, population characteristics can vary widely and result in a wide range of depression severity. Patients with more severe depression seem to have a higher risk of stroke, possibly independent of age effects [11]–[13]. Thirdly, most studies fail to provide information on antidepressant treatment and outcome of depression treatment. Antidepressant treatments may improve stroke outcome [14], [15], whereas certain antidepressants (i.e., selective serotonin reuptake inhibitors [SSRIs]) may increase bleeding tendency by inhibiting platelet aggregation and have been associated with higher risk of stroke [16], [17]. A systematic review of the literature also found the positive association of medication use with stroke risk [10]. However, the observed drug-stroke association may be confounded by factors such as the severity of depression at presentation and an identified history of clinical depression [10], [16]–[19].

Therefore, in the present study, we aimed to test two hypotheses, using data from the nationwide database of the National Health Insurance (NHI) Program in Taiwan (1996–2009). Firstly, since depression is more likely to develop and be comorbid with the aforementioned stroke risk factors, such as HTN and hyperlipidemia [20], [21], we hypothesized that a history of clinical depression would not directly increase the risk for stroke, but indirectly through the known stroke risk factors such as major metabolic diseases. Secondly, since depressive symptoms are a risk factor strongly associated with stroke [9], [10], we hypothesized that more severe depressive symptoms, not antidepressant prescriptions, are associated with stroke onset.

## Materials and Methods

### Data Sources

We used the 1996–2009 NHI database to conduct this population-based matched cohort study, which was published by the National Health Research Institute (NHRI) of Taiwan. The coverage rate of the NHI program for all its residents was elevated to 99% at the end of 2004. The database comprises comprehensive information about clinical visits for each insured subject, including diagnostic codes according to the clinical modification of the International Classifications of Disease-9 (ICD-9-CM) and prescription details [22]. Since the dataset was released for research purposes and included only scrambled information on patient and physician identification, the study was exempt from full review by the local Ethics Review Committee.

### Study Subjects

**Figure 1:**
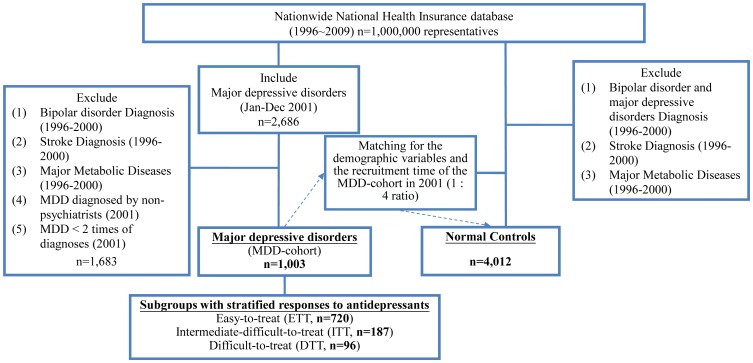
Flow diagram of sample selection.

Our study cohort (MDD-cohort) included all patients who had at least 2 diagnoses of MDD (ICD-9-CM code: 296.2 and 296.3) made by psychiatrists from January to December 2001 from the sample of 1 million individuals randomly selected from the 23 million beneficiaries of the NHI scheme of Taiwan (Figure 1), using criteria identical to that in our previously published paper [23]. To prevent misdiagnosis of major depression (i.e., a selection bias), we excluded patients diagnosed with bipolar disorders and affective psychosis between 1996 and 2000. A group of normal subjects (a 1∶4 ratio = 1 MDD subject to 4 well-matched normal subjects) matched with the corresponding MDD patients in the MDD cohort in terms of demographic variables (i.e., age and gender) and recruitment time, as well as not having a MDD or BD diagnosis before recruitment, acted as controls. All subjects had no major metabolic diseases or stroke before recruitment, using the criteria described in the following sections. Other recruitment details were available online (supplementary materials).

### Definitions of Stroke and Stroke-related Factors During Whole Study Period

#### Stroke and major metabolic diseases (diabetes mellitus, hypertension and hyperlipidemia)

Stroke was identified using ICD-9-CM codes 430.X-438.X for cerebrovascular events, which cover hemorrhagic, ischemic and other types of stroke, to maximize the identification of potential stroke events. Diabetes mellitus (DM), HTN and hyperlipidemia were identified only when the criteria of a respective diagnosis of an ICD-9-CM code and a corresponding prescription of drugs were both fulfilled. Detailed information on the diagnostic codes and drugs was available online (supplementary materials).

#### Depression severity: baseline levels of refractoriness and pre-stroke depressive symptoms

To investigate the associations between depression severity and stroke, depressive severity was approached from 2 different dimensions – baseline levels of refractoriness (i.e., a trait-like characteristic) and an estimation of pre-stroke depressive symptoms (i.e., state-like).

One dimension that we measured depressive severity of the MDD patients was levels of refractoriness to antidepressants. Such assessment was based on a stratification method described in detail before [23]. We categorized depressives into 3 groups based on their baseline response to antidepressants (2000–2002) – i.e., ETT (easy to treat), ITT (intermediate difficult to treat) and DTT (difficult to treat). DTT patients were defined as those patients who were shifted from adequate antidepressant trials 2 or more times. An adequate trial was defined by the use of an antidepressant within its therapeutic dosage range (e.g., fluoxetine > = 20 mg/day) for more than 60 days consecutively. The ETT groups included those patients maintaining antidepressants and those without antidepressant prescriptions. Finally, the ITT group was composed of those who changed their antidepressants once only. During the follow-up period (2001–2009), the frequency of emergency room (ER) visits, psychiatric hospitalizations and non-psychiatric hospitalizations for all subjects and the percentage of patients having attempted suicide were also identified.

The other dimension about depressive severity was levels of time-dependent depressive symptoms. To explore the level of pre-stroke depressive symptoms in the patients with stroke, another well-matched depression group were selected within the MDD cohort and average psychiatric visits during the 6-month period before stroke onset were analyzed. Since specialists are easy to reach in Taiwan, patients with more psychiatric visits in a given period were assumed to have experienced higher levels of depressive symptoms.

#### Other independent variables – psychiatric and substance comorbidities

Dysthymia, anxiety (anxiety states and anxiety disorders) and substance abuse or dependence, such as alcoholism and nicotine abuse and dependence, are frequently comorbid with major depression. These comorbidities were also studied to avoid a confounding effect on stroke risk. Definitions of psychiatric comorbidity and substance abuse/dependence were available online (supplementary materials).

#### Associations between antidepressant drugs and stroke

We retrospectively reviewed the antidepressant prescription patterns of depressed patients suffering from stroke during the follow-up period. The presence of any antidepressant prescriptions 90 days before the onset of stroke in the depressed patients was defined as medicated; otherwise, they were regarded as un-medicated. If a stroke incident occurred 14 days after the initiation of new antidepressants, we reasoned that the stroke could be related to the prescribed antidepressant by the fact of the close temporal relationship between the antidepressant and the stroke incidence. We also checked the antidepressant prescription patterns in the 28 days before the stroke incidences, mainly due to the concern about the anti-platelet prosperities associated with certain antidepressants like selective serotonin reuptake inhibitors [24].

### Statistics

The SAS statistical package (SAS System for Windows, version 9.2; SAS Institute, Cary, NC, U.S.A.) and SPSS statistics (SPSS for Windows, version 18·0, Microsoft Corporation, Chicago, IL, U.S.A.) were used to perform the data analysis. Categorical variables between groups were analyzed by chi-square test (or Fisher’s exact test if less than 5 cases in any given group). Continuous variables between groups were analyzed by independent *t*-tests or the one-way ANOVA test.

To investigate whether a history of clinical depression would increase the risk for stroke directly or indirectly through the mediation of major metabolic diseases, mediation regression analyses were conducted using the procedures outlined by Baron and Kenny [25]. We first conducted simple regression analyses to establish whether significant relationships exist between depression and major metabolic diseases, between depression and stroke, and between major metabolic diseases and stroke. Then, to test whether the major metabolic disease is an important intervening variable, a multiple regression analysis with depression and major metabolic diseases (i.e., the intervening variable) predicting stroke was conducted. Odd ratios (OR) with 95% confidence interval (CI) for OR were reported. Some form of mediation is supported if the effect of major metabolic diseases was significant after controlling for depression. If depression is no longer significant when the intervening variable of the major metabolic diseases is controlled, a full mediation is supported [25]. The relationships between psychiatrist-diagnosed clinical depression (i.e., the MDD cohort and controls) at baseline and the subsequent development of stroke (from 2001 to 2009) were also examined using Cox-PH regression models, with and without adjusting baseline (e.g., sex and age) and time-dependent factors (e.g., major metabolic and substance comorbidities).

To study whether stroke incidences happened in a more refractory MDD subgroup (i.e., DTT and ITT vs. ETT), we later replaced the factor of clinical MDD diagnosis with MDD subgroups into the Cox-PH regression model. All assumptions for the Cox models were tested and met. Beta values (β), standard errors (S.E.), and hazard ratios (HR, as a measure of effect size) and their 95% confidence intervals (CI) were all reported. P<0.05 (2-sided tests) was deemed to be statistically significant.

## Results

### Demographic/clinical Variables/stroke between Depression and Normal Subjects

**Figure 2:**
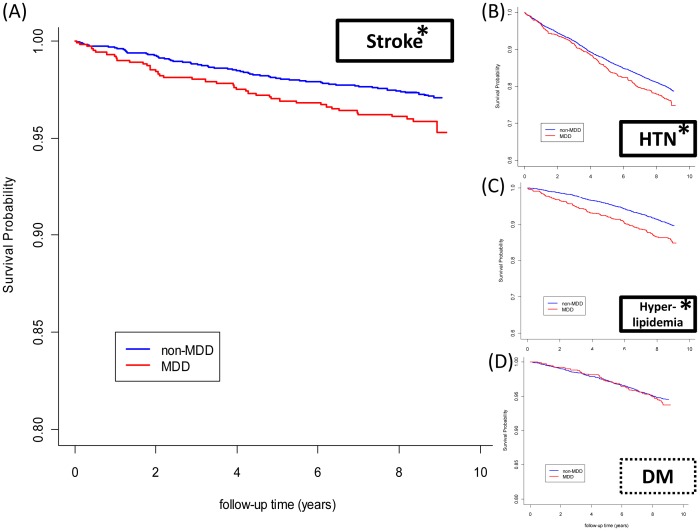
Kaplan Meier survival plots for MDD and non-MDD subjects. Patients with MDD (blue lines) were more likely to suffer from stroke (A), HTN (B), hyperlipidemia (C), but not DM (D) over time than normal subjects (red lines). Axis-X represents the time in years, and axis-Y the percentage survival. A log-rank test was used to compare the between-group survival distributions and a value of p<0.05 was considered statistically significant.

One thousand three patients (MDD cohort) and 4012 well-matched subjects (control group) were recruited and no missing subject was established during the 9-year follow-up. MDD patients had significantly more ER visits, psychiatric hospitalizations and suicidal attempts than the controls (see the lower panel in Table 1). During the 9-year follow-up period, MDD patients also had more chances in non-psychiatric hospitalizations, stroke incidences (Figure 2A), and the development of HTN (Figure 2B) and hyperlipidemia (Figure 2C) than the controls, but not in DM (Figure 2D).

**Table 1:** Demographic data, clinical variables and comorbidities of major metabolic diseases and stroke in depressed patients and normal controls.

	Groups	N	Age (years)Mean±SD	Male N (%)	ER visits a (times)Mean±SD	PsychiatrichospitalizationsN (%) [times,Mean±SD]	SuicidalattemptsN (%)	Non-psychiatrichospitalizationsN (%)	StrokeN (%) [years,Mean ± SD]	HTN N (%)[years,Mean ± SD]	Hyper-lipidemiaN (%)	DMN (%)
MDD	ETT	720	40.82±14.80	266 (36.9)	2.17±4.51	96 (13.3) [0.97±5.77]	28 (3.9)	189 (26.3)	34 (4.7) [3.88±2.77]	174 (24.2) [4.06±2.61]	91 (12.6) [4.30±2.75]	35 (4.9) [5.17±2.58]
	ITT	187	42.39±14.45	63 (33.7)	3.02±4.66 **^§^**	46 (24.6) [1.11±3.41]	12 (6.4)	66 (35.3)	7 (3.7) [3.40±2.42]	43 (23.0) [4.80±2.41]	33 (17.6) [4.20±2.31]	19 (10.2)[4.83±2.31]
	DTT	96	41.87±12.18	38 (39.6)	3.66±5.75**^Φ,^** ^ ¶^	35 (36.5) [2.82±8.62]	10 (10.4)	32 (33.3)	2 (2.1) [1.38±0.56]	22 (22.9) [3.03±1.60]**^?,?^**	19 (19.8) [4.87±2.39]	6 (6.2) [6.39±1.76]
*p*-value (ETT vs. ITT vs. DTT)			0.369 b	0.588 c	**0.009** b, **	**<0.001** c, ** [0.124 b]	**0.016** c, *	**0.028** c, *	0.423 c [0.179 b]	0.852 c **[0.003** b,** **]**	0.075 c, †[0.636 b]	**0.026** c, * [0.395 b]
All MDD		1,003	41.22±14.51	367 (36.6)	2.47±4.69	177 (17.7) [1.17±5.78]	50 (5.0)	287 (28.6)	43 (4.3) [3.68±2.68]	239 (23.8) [4.10±2.53]	143 (14.3) [4.35±2.60]	60 (6.0) [5.19±2.43]
Normal Controls		4,012	41.20±14.50	1,468 (36.6)	0.99±4.35	44 (1.10) [0.09±1.68]	14 (0.3)	628 (15.7)	113 (2.8) [3.88±2.57]	819 (20.4) [4.04±2.54]	395 (9.8) [5.08±2.42]	214 (5.3) [4.68±2.45]
*p*-value (MDD vs. Controls)			0.977 d	>0.999 c	**<0.001** d, **	**<0.001** c, ** [0.124 d]	**<0.001** c, **	**<0.001** c, **	**0.015** c, * [0.673 d]	**0.016** c, *[0.771 d]	**<0.001** c, ** [0.003 d,*]	0.419 c [0.163 d]

Note. ER, emergency room; HTN, hypertension; DM, diabetes mellitus.

a Rankings of ER visits for affective problems: 2^nd^ in ETT, 1^st^ in ITT and 1^st^ in DTT.

b ANOVA analysis, post-hoc (LSD): **^Φ^**DTT > ETT, ^¶^ DTT > ITT, **^§^** ITT > ETT,**^¥^**DTT < ETT,**^<$>\vskip -2pt\scale 60%\raster="rg2"<$>^**DTT < ITT.

c Chi-square test.

d Independent *t*-test.

* p<0.05,

** p<0.005,

† p<0.10 (values in bold: p<0.05).

### Demographic/clinical Variables/stroke Among Depression Groups (i.e., ETT, ITT and DTT)

**Figure 3:**
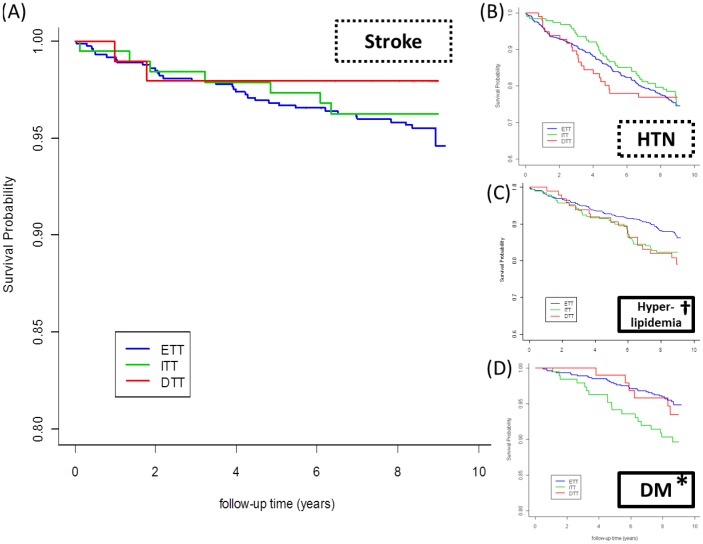
Kaplan Meier survival plots among MDD patients with stratified responses to antidepressants. DTT patients (red lines) did not differ from ITT (green lines) or ETT (blue lines) patients with respect to stroke (A) and HTN (B), but trended higher in developing hyperlipidemia (C) and had significantly more risks of developing DM (D) over time. Axis-X represents the time in years, and axis-Y the percentage survival. A log-rank test was used to compare the between-group survival distributions and a value of p<0.05 was considered statistically significant.

The sex [female/male ratio, ranged from 1.53 (DTT) to 1.96 (ITT)] and age (all: mean+/−SD = 41.22+/−14.51) characteristics of these 3 groups did not differ statistically (Table 1). The greater trait-like depressive severity in the DTT group was further corroborated by the findings of significantly more ER visits for psychiatric problems (p<0.009), more psychiatric hospitalizations (p<0.001) and greater chances of attempting suicide (p<0.05) than in the ITT and the ETT group over the follow-up period. There were no significant between-group differences in stroke rates (ETT vs. ITT vs. DTT = 4.7% vs. 3.7% vs. 2.1%, p<0.05) and the comorbidity of HTN (24.2% vs. 23.0% vs. 22.9%, p<0.05) during the follow-up (Figures 3A &3B). However, the ETT group had a relatively lower chance of developing hyperlipidemia (p<0.10, a trend significance) and DM (p<0.05) (Figures 3C & 3D), as well as a lower non-psychiatric hospitalization rate (p<0.05).

### Clinical Depression on Stroke Risks after Adjusting Major Covariates

To establish whether significant relationships exist among depression, major metabolic diseases and stroke, separate regression analyses were conducted. We found that the odd ratios (OR) were all significant [OR (95% CI for OR) for depression-metabolic diseases: 1.546(1.080–2.211), p = 0.017; depression-stroke: 1.366 (1.177–1.587), p<0.001; metabolic diseases-stroke: 8.496 (5.880–12.276), p<0.001] (Table 2). Then, a multiple regression analysis with depression and major metabolic diseases predicting stroke was conducted. We found that the OR for depression predicting stroke was no longer significant (1.344 (0.932–1.938), p = 0.114), whereas the effect of major metabolic diseases predicting stroke remains significant (OR = 8.367 (5.788–12.096), p<0.001). Taken together, the findings supported our hypothesis of a full mediation effect leading depression to stroke through major metabolic diseases.

**Table 2:** Mediation analysis showing the development of major metabolic diseases as a mediator leading psychiatrist-diagnosed major depressive disorder (MDD) to subsequent stroke incidences in 5015 Taiwan inhabitants, 2001–2009.

Mediation regression analyses		Variable	β(S.E)	O.R (95% ConfidenceInterval)	*p*
Step 1	MDD diagnosis → Stroke	MDD diagnosis	0.435 (0.183)	1.546 (1.080 to 2.211)	**0.017** *
Step 2	MDD diagnosis → Major metabolic diseases	MDD diagnosis	0.312 (0.076)	1.366 (1.177 to 1.587)	**<0.001** *
Step 3	Major metabolic diseases → Stroke	Major metabolic diseases	2.140 (0.188)	8.496 (5.880 to 12.276)	**<0.001** *
Step 4	MDD diagnosis + Major metabolic diseases → Stroke	MDD diagnosis	0.295 (0.187)	1.344 (0.932 to 1.938)	0.114
		Major metabolic diseases	2.124 (0.188)	8.367 (5.788 to 12.096)	**<0.001** *

* *p*<0.05, statistically significant.

The Cox-PH regression model showed that the HR associated with MDD diagnosis was 1.544 (p<0.015), after adjustments for age and sex (Model I, Table S2). In the models that included additional adjustments for major metabolic diseases or substance abuse/dependence, MDD diagnosis was still associated with a 43.9–47.1% increased risks of developing stroke over time (Model II and III, Table S2). However, if adjusting for age, sex, major metabolic diseases, and substance comorbidities, the HR (1.380) associated with MDD diagnosis failed to reach a statistical significance (p>0.05) (Model IV, Table S2), implicating that clinical depression diagnosis increased stroke risks indirectly through a development of other major stroke-related factors such as hypertension, diabetes and substance abuse/dependence. The stroke HRs associated with the DTT group (ETT group used as a reference) was 0.460 (0.109 to 1.946, p = 0.369) and with the ITT group was 0.685 (0.300 to 1.565, p = 0.291) after adjusting age, sex, hypertension, hyperlipidemia, diabetes, psychiatric and substance comorbidities.

### Temporal Relationships between Stroke and Major Comorbidities

Most patients with stroke (33/43, 76.7%) had comorbidities with major metabolic diseases, yet only very few of them (4/43, 9.3%) had comorbidities with substance abuse/dependence and all of these 3 subjects were in the ETT groups and all developed substance problems before stroke onset. Therefore, we further investigated the temporal relations between major metabolic diseases and stroke (Table 3).

**Table 3:** Temporal relationship between stroke and major metabolic diseases in 3 depression groups with stratified responses to antidepressants.

Groups	Stroke & HTN		Stroke & Hyperlipidemia		Stroke & DM		Stroke & All a	
	HTN first	Stroke first	Hyperlipidemia first	Stroke first	DM first	Stroke first	All a first	Stroke first
ETT b	12 (60)	8 (40)	4 (57.1)	3 (42.9)	5 (100)	0 (0.0)	19 (76)**	6 (24)
ITT b	2 (40)	3 (60)	1 (33.3)	2 (66.7)	1 (33.3)	2 (66.7)	3 (50)	3 (50)
DTT b	1 (50)	1 (50)	0 (0.0)	0 (0.0)	0 (0.0)	0 (0.0)	1 (50)	1 (50)
*p*-value c		0.714		0.490		0.035*		0.829

Note. Data shown as N (%); HTN, hypertension; DM, diabetes mellitus.

a All = concurrent hypertension, hyperlipidemia and diabetes mellitus.

b Within-group analysis by chi-square.

c Between-group analysis by fisher’s exact tests.

* p<0.05,

** p<0.005.

The ETT patients mostly developed major metabolic diseases before stroke. The healthy subjects also demonstrated a similar pattern of developing major metabolic diseases before the onset of stroke (84/113, 74.3%). There was no difference in the distribution between the normal subjects and the ETT patients (p = 0.863). However, this pattern could not be observed in the ITT and the DTT groups. The mean +/−SD duration in years to develop stroke was the shortest among patients with DTT (1.38+/−0.56) relative to the other 2 groups (despite a lack of statistical significance).

### Associations between Antidepressants and Stroke

In the period before stroke onset (14 days before stroke), we found that around half (48.8%) of the stroke patients were un-medicated and also that another half (48.8%) of the stroke patients did not change their antidepressants from what had been prescribed 3 months before. Only one subject was prescribed a new antidepressant (bupropion, 150 mg/day) on the 14^th^ day before the stroke incident (Table S1). Likewise, if we reexamined the antidepressant prescription patterns in the 28 days before the stroke incidences, we found a similar pattern and there was still a high proportion of patients who did not use antidepressants before stroke (19/43, 44.2%) or did not change antidepressants (21/43, 48.9%), whereas only 7.0% (3/43) of patients had switched their antidepressant to a new one. In addition to the one prescribed with bupropion (150 mg/day) on the 14^th^ day before the stroke incident, the other 2 patients were respectively prescribed with venlafaxine (75 mg/d) and fluoxetine (20 mg/d) on the 28^th^ day before the stroke incident. All of these 3 subjects suffered from ischemic but not hemorrhagic strokes (i.e., 2 diagnosed with ICD9 code 434 and 1 with 435). Because the temporal relationships between antidepressant prescriptions and strokes could be a reflection of exacerbated depressive symptoms and the fact that no patient on recent antidepressants suffered from hemorrhagic stroke, we believed the concern about the link between SSRI antidepressants (due to their anticoagulant properties) and hemorrhagic strokes could be minimized in the clinical settings. These results confirmed that there were no clinically relevant associations between antidepressants and stroke.

### Pre-stroke Depressive Symptom Levels on Stroke Risks

To investigate the depressive symptom levels before the onset of stroke and to retrospectively examine whether the refilling pattern with unchanged antidepressants (unchanged antidepressants: N = 21, Table S1) was appropriate, 44 well-matched depressed patients (matched for age, gender, drug-refractoriness group and the time of stroke with the corresponding 22 stroked individuals under medication control, in a 2∶1 ratio) were recruited. We found the average number of psychiatric visits of MDD patients 6 months before the onset of stroke was significantly higher for the stroked than the controlled depressive subjects (mean +/− SD: 5.36+/−3.92 vs. 2.66+/−4.07 times, p = 0.0123), indicating that the level of depressive symptoms was enhanced in MDD patients right before stroke.

## Discussion

### Strengths of the Study and Summaries of Main Findings

The strengths of the current study are the large sample size, the long follow-up period (2001–2009), the recruitment of all incident stroke cases and, most importantly, the diagnosis of clinical MDD by specialists in all cases. Furthermore, many previous studies focused on a selected population (e.g., older or elderly people), instead of a general population as is the case in the present study. By using this database covering a representative cohort of 1,000,000 enrollees (1996–2009), we confirmed that a history of MDD increased stroke risk, yet in an indirect way such as through higher levels of depressive symptoms and the development of major metabolic and/or substance comorbidities. However, no increase in stroke risk in patients showing greater refractoriness to antidepressants, and no clinical association between antidepressant use and the development of stroke, were found. Notably, compared with refractory patients and healthy subjects, patients with greater refractoriness to antidepressant therapy developed major metabolic diseases (e.g., HTN) less frequently. Key factors linking depression to stroke are summarized in supplementary figure 1.

### Levels of Depressive Symptoms and Antidepressants before Stroke Onset

It has never been easy to evaluate the impact of depressive symptoms right before stroke in a long-term follow-up study. This is because stroke occurs suddenly or it occurs one day several years later, and less than 10% of depressed patients suffer from stroke. In the present study, the level of depressive symptoms was significantly greater before the onset of stroke. However, stroke risk did not increase in patients with greater refractoriness to antidepressant treatment. We believe this observation reflects a protective effect of long-term antidepressant use, since antidepressant treatment reduced stroke mortality significantly [14]. Also, no clinical evidence links antidepressant use to the development of stroke. Our results support the previous observation of a close relationship between the use of antidepressants and stroke events. Greater depression severity preceding stroke onset and/or long-term poor adherence to psychiatric treatment may reflect this relationship. This notion could be further supported by the finding of a negative correlation between stroke risk and the number of antidepressants used [26].

### Temporal Relations between Comorbidities and Strokes and Clinical Implications

A differential pattern of associations between major comorbidities and strokes was demonstrated in refractory and non-refractory MDD. The patients with less trait-like impairment (i.e., relatively easy-to-treat patients) were more likely to suffer from HTN, DM, hyperlipidemia, and substance problems before stroke onset. HTN is one of the most important modifiable factors linked to later stroke in the general population [3], [8]. Our study increases the generalizability of the findings to depressed populations. We found that a large proportion of depressed patients suffered from major comorbidities first, most frequently HTN (12/19 = 63.2%; Table 2) before stroke onset. We also demonstrated that the temporal relation between major metabolic comorbidities and stroke was similar in healthy subjects. One of the underlying mechanisms linking depression and HTN may be activation of the hypothalamus-pituitary-adrenal axis. Negative emotions are processed through the amygdala [27], [28], and the corresponding signals result in activation of the stress circuit via the hypothalamus-pituitary-adrenal axis [29]. Persistently elevated adrenalin levels due to chronic and prolonged HPA activation will eventually lead to a fragile cerebrovascular vasculature and the development of hypertension [30], [31]. We found that patients with greater trait-like impairment (i.e., the difficult-to-treat patients) more frequently bypassed this stage and developed strokes sooner, which further supports the effect of worsened depressive symptoms on stroke risk. It is plausible that cerebrovascular vasculature of DTT patients gradually becomes more fragile and that episodic surges of high blood pressure directly increase the likelihood of a stroke event. Our findings imply that in addition to treatment of depression, another pivotal strategy for national stroke prevention should be prevention and treatment of HTN.

### Limitations

There were some limitations that should be addressed. Firstly, unhealthy lifestyle (e.g., physical activity and dietary intake) could contribute to obesity and overweight and thereby to higher risk for stroke [32], yet this registry-based database does not contain such information. However, it can be expected that even if a thorough obesity profile were entered into the regression model and the depression-stroke association was further attenuated, the conclusions drawn from the present analysis would not be changed. Secondly, keeping database records of some variables (e.g., suicide attempts) is not officially mandated, and therefore the incidence could be somewhat underestimated. However, this kind of information bias applies to each MDD subgroup and our aim was not to study the incidence. Finally, different kinds of stroke events (e.g., hemorrhagic or ischemic) were all identified to maximize stroke incidence and to reduce potential selection bias. Since each kind of event represents a relatively small proportion of the total number of stroke events, we did not conduct further analysis.

### Conclusion

A clinical diagnosis of major depression was indirectly associated with higher risk of future stroke. Stroke risk is increased by higher levels of depressive symptoms and the development of major metabolic and substance comorbidities, but not by a history of refractoriness to antidepressants and the prescription of antidepressant drugs.

## Supporting Information

Figure S1 **A schematic diagram showing major stroke-related factors in major depression.** Depression severity could be divided into levels of antidepressant refractoriness and depressive symptoms. The most important stroke-related factors (solid red arrows) include a comorbidity of substance abuse/dependence and major metabolic diseases and higher levels of depressive symptoms. Levels of refractoriness and antidepressants were not associated with higher stroke risks over time (solid black arrows). Non-refractory patients, for the most part, developed stroke after the development of major metabolic diseases, whereas refractory ones developed stroke in a more direct way.
(TIF)

Table S1 **Retrospective review of antidepressant prescription patterns before the onset of stroke in the depressed patients.** ^a^Un-medicated or medicated: defined by the presence of any antidepressant prescriptions 3 months before stroke. ^b^Unchanged or new: defined by the prescribed antidepressants 14 days before stroke, compared to the one prescribed 14 to 90 days before stroke. ^c^ this patient had no antidepressant prescription (14–90 days before stroke), but was prescribed bupropion on the 14^th^ day before stroke.
(DOCX)

Table S2 **Relation between psychiatrist-diagnosed major depressive disorder (MDD) and stroke incidences with and without major stroke-related covariates in 5015 Taiwan inhabitants, 2001–2009.** * *p*<0.05, statistically significant.
(DOCX)

Text S1 (DOCX)
