# Supplementary figures and images for: Major Depressive Disorder and Stroke Risks: A 9-Year Follow-Up Population-Based, Matched Cohort Study
Source: PLoS One. 2012 Oct 8;7(10):e46818. doi: 10.1371/journal.pone.0046818 (PMC3466174; doi:10.1371/journal.pone.0046818)

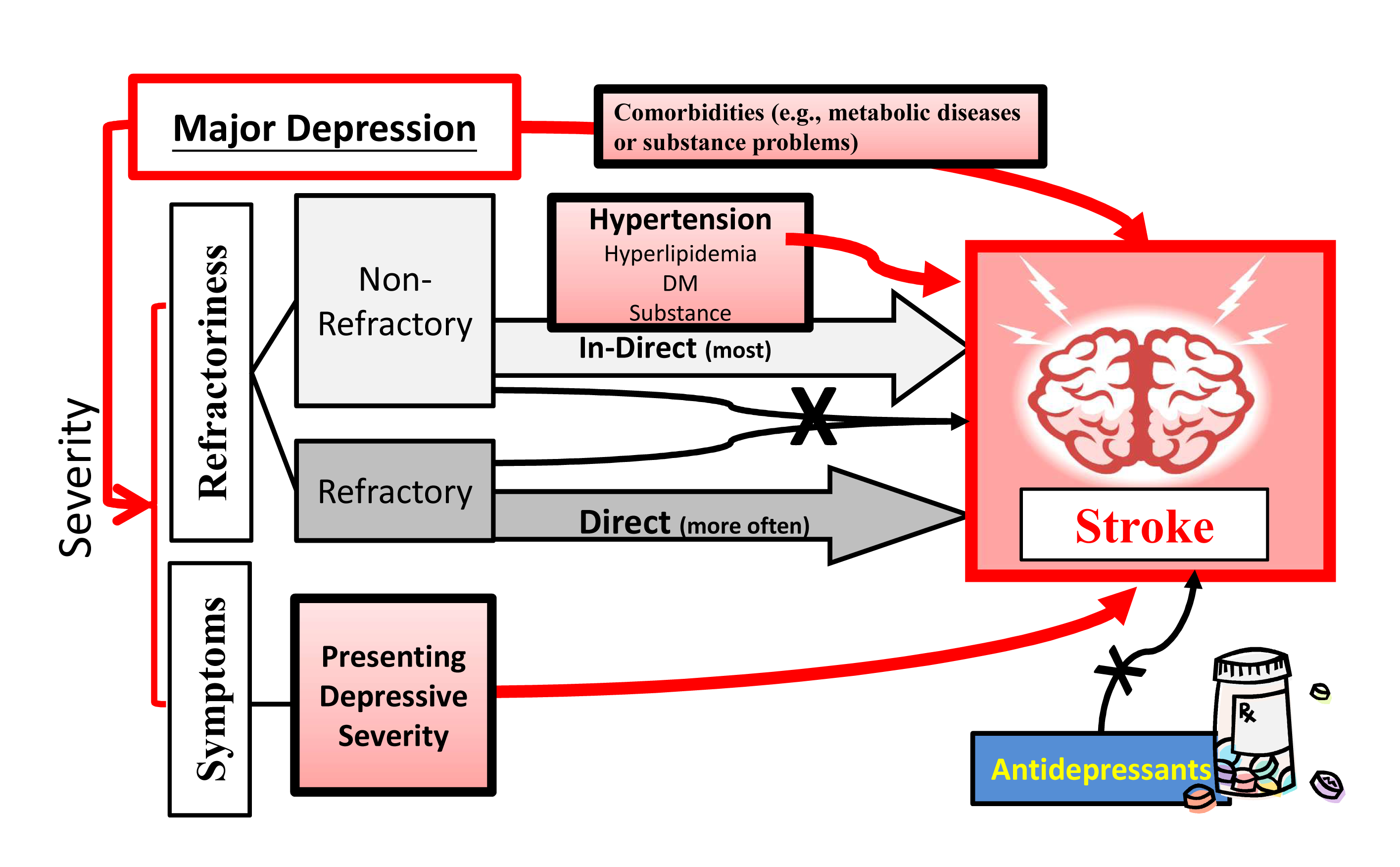

Supplement: Figure S1 — A schematic diagram showing major stroke-related factors in major depression. Depression severity could be divided into levels of antidepressant refractoriness and depressive symptoms. The most important stroke-related factors (solid red arrows) include a comorbidity of substance abuse/dependence and major metabolic diseases and higher levels of depressive symptoms. Levels of refractoriness and antidepressants were not associated with higher stroke risks over time (solid black arrows). Non-refractory patients, for the most part, developed stroke after the development of major metabolic diseases, whereas refractory ones developed stroke in a more direct way. (TIF) [file pone.0046818.s001.tif]
