# Supplementary material for: Major Depressive Disorder and Stroke Risks: A 9-Year Follow-Up Population-Based, Matched Cohort Study
Source: PLoS One. 2012 Oct 8;7(10):e46818. doi: 10.1371/journal.pone.0046818 (PMC3466174; doi:10.1371/journal.pone.0046818)
Supplement: Table S1 — Retrospective review of antidepressant prescription patterns before the onset of stroke in the depressed patients. aUn-medicated or medicated: defined by the presence of any antidepressant prescriptions 3 months before stroke. bUnchanged or new: defined by the prescribed antidepressants 14 days before stroke, compared to the one prescribed 14 to 90 days before stroke. c this patient had no antidepressant prescription (14–90 days before stroke), but was prescribed bupropion on the 14th day before stroke. (DOCX) [file pone.0046818.s002.docx]

**Table S1.** Retrospective review of antidepressant prescription patterns before the onset of stroke in the depressed patients

| Groups | N | Un-medicated ^a^  (N=21) | Medicated ^a^ (N=22) | |
| --- | --- | --- | --- | --- |
|  |  |  | Unchanged Antidepressants ^b^  (N=21) ^b^ | New  Antidepressants ^b^  (N=1) |
| ETT | 34 | 17 (80.95%) | 17 (80.95%) | 0 (0.00%) |
| ITT | 7 | 4 (19.05%) | 2 (9.52%) | 1 (100%) ^c^ |
| DTT | 2 | 0 (0.00%) | 2 (9.52%) | 0 (0.00%) |

^a^ Un-medicated or medicated: defined by the presence of any antidepressant prescriptions 3 months before stroke

^b^ Unchanged or new: defined by the prescribed antidepressants 14 days before stroke, compared to the one prescribed 14 to 90 days before stroke

^c^ this patient had no antidepressant prescription (14 - 90 days before stroke), but was prescribed bupropion on the 14^th^ day before stroke
