# Supplementary material for: Major Depressive Disorder and Stroke Risks: A 9-Year Follow-Up Population-Based, Matched Cohort Study
Source: PLoS One. 2012 Oct 8;7(10):e46818. doi: 10.1371/journal.pone.0046818 (PMC3466174; doi:10.1371/journal.pone.0046818)
Supplement: Table S2 — Relation between psychiatrist-diagnosed major depressive disorder (MDD) and stroke incidences with and without major stroke-related covariates in 5015 Taiwan inhabitants, 2001–2009. * p<0.05, statistically significant. (DOCX) [file pone.0046818.s003.docx]

**Table S2.** Relation between psychiatrist-diagnosed major depressive disorder (MDD) and stroke incidences with and without major stroke-related covariates in 5015 Taiwan inhabitants, 2001-2009

| Model | Covariates included | β(S.E) | H.R (95% Confidence Interval) | *p* |
| --- | --- | --- | --- | --- |
| I | MDD diagnosis, age and sex | 0.435 (0.179) | 1.544 (1.087 to 2.194) | **0.015*** |
| II | MDD diagnosis, age, sex, and major metabolic diseases | 0.386 (0.179) | 1.471 (1.035 to 2.091) | **0.031*** |
| III | MDD diagnosis, age, sex, and substance abuse/dependence | 0.364 (0.183) | 1.439 (1.005 to 2.061) | **0.047*** |
| IV | MDD diagnosis, age, sex, major metabolic diseases, and substance | 0.322 (0.184) | 1.380 (0.963 to 1.987) | 0.080 |

* *p* < 0.05, statistically significant
