# Supplementary material for: Major Depressive Disorder and Stroke Risks: A 9-Year Follow-Up Population-Based, Matched Cohort Study
Source: PLoS One. 2012 Oct 8;7(10):e46818. doi: 10.1371/journal.pone.0046818 (PMC3466174; doi:10.1371/journal.pone.0046818)
Supplement: Text S1 — (DOCX) [file pone.0046818.s004.docx]

**Supporting Information Legends_Text**

**Other details for study subjects**

To prevent misdiagnosis of major depression (i.e., a selection bias), we excluded patients diagnosed with bipolar disorders (ICD-9-CM code: 296.0, 296.1, 296.4, 296.5, 296.6, 296.7 and 296.8), and affective psychosis (ICD-9-CM code: 296.9) between 1996 and 2000. To further ensure the validity of the diagnosis of MDD, we excluded patients whose diagnosis of MDD was not made by psychiatrists and included only those having at least 2 diagnoses of MDD made by psychiatrists.

**Other details for definitions of stroke and stroke-related factors**

***Definitions of stroke and major metabolic diseases***

Stroke covers hemorrhagic stroke (ICD-9-CM code 430.X, 431.X and 432.X), ischemic stroke (ICD-9-CM code 433.X, 434.X and 435.X) and other stroke (ICD-9-CM code 436.X, 437.X and 438.X). Diabetes mellitus (DM) was identified only when a diagnosis of ICD-9-CM code 250.X and a prescription of drugs for DM were both fulfilled. Likewise, hypertension (HTN) and hyperlipidemia were identified only when a diagnosis of ICD-9-CM code (HTN: 401.X-405.X; and hyperlipidemia: 272.X) and a prescription of corresponding drugs were both fulfilled. Anatomical Therapeutic Chemical (ATC) codes will be used for the identification of the prescription drugs. Drugs for DM included ATC code A10A (insulins and analogues), A10B (oral blood glucose lowering drugs). Drugs for HTN included C01A (cardiac glucosides), C01B (antiarrhythmics, class I and III), C01C (cardiac stimulants excl. cardiac glucosides), C01D (vasodilators used in cardiac diseases), C01E (other cardiac preparations), C02 (antihypertensives),C03 (diuretics), C04 (peripheral vasodilators), C05 (vasoprotectives), C07 (beta blocking agents) and C08 (calcium channel blockers), and C09 (agents acting on the renin-angiotensin system); however, propranolol (ATC code: C07AA05) and atenolol (ATC code: C07AB03) were excluded due to the fact that they are commonly used for treating depression-related anxiety symptoms. Drugs for hyperlipidemia included C10 (lipid modifying agents).

***Definitions for psychiatric comorbidity and substance abuse/dependence***

Dysthymia (ICD-9-CM code: 300.4), anxiety states (300.0, 300.00, 300.09, 300.1, 300.15, 300.8, 300.89 and 300.9), anxiety disorders including panic disorder (300.01), agoraphobia with panic attacks (300.21), generalized anxiety disorder (300.02), social phobia (300.23), obsessive-compulsive disorders (300.3), neurasthenia (300.5), hypochondriasis (300.7) and somatization disorder (300.81) were identified. Regarding substance abuse and dependence, alcohol abuse and dependence (303, 303.9, 303.9X, 305.0X) as well as other substance abuse and dependence such as nicotine (305.1X), opioid (304.0X & 305.5X), barbiturate (304.1X & 305.4X), cocaine (304.2X & 305.6X), cannabis (304.3X & 305.2X), amphetamine (304.4X & 305.7X) and hallucinogen (304.5X & 305.3X) were also identified for all recruitment subjects.

**Figure S1**. **A schematic diagram showing major stroke-related factors in major depression.** Depression severity could be divided into levels of antidepressant refractoriness and depressive symptoms. The most important stroke-related factors (solid red arrows) include a comorbidity of substance abuse/dependence and major metabolic diseases and higher levels of depressive symptoms. Levels of refractoriness and antidepressants were not associated with higher stroke risks over time (solid black arrows). Non-refractory patients, for the most part, developed stroke after the development of major metabolic diseases, whereas refractory ones developed stroke in a more direct way.
